# Fabrication of Stretchable Copper Coated Carbon Nanotube Conductor for Non-Enzymatic Glucose Detection Electrode with Low Detection Limit and Selectivity

**DOI:** 10.3390/polym10040375

**Published:** 2018-03-28

**Authors:** Dawei Jiang, Zhongsheng Liu, Kunkun Wu, Linlin Mou, Raquel Ovalle-Robles, Kanzan Inoue, Yu Zhang, Ningyi Yuan, Jianning Ding, Jianhua Qiu, Yi Huang, Zunfeng Liu

**Affiliations:** 1 School of Materials Science and Engineering, Jiangsu Collaborative Innovation Center of Photovolatic Science and Engineering, Changzhou University, Changzhou 213164, China; 18652856837@163.com (D.J.); wukunkun1030342792@163.com (K.W.); nyyuan@cczu.edu.cn (N.Y.); dingjn@cczu.edu.cn (J.D.); jhqiu@cczu.edu.cn (J.Q.); 2 State Key Laboratory of Medicinal Chemical Biology, Key Laboratory of Functional Polymer Materials, Ministry of Education, College of Pharmacy, Nankai University, Tianjin 300071, China; 13222539532@163.com (Z.L.); 13672133169@163.com (L.M.); yihuang@nankai.edu.cn (Y.H.); 3 Lintec of America, Nano-Science and Technology Center Richardson, Dallas, TX 75081, USA; raquel-ovalle@lintec-nstc.com (R.O.-R.); kz-inoue@lintec-nstc.com (K.I.); 4 Department of Building Engineering, Logistics University of PAPF, Tianjin 300309, China; zhangyu1983xinxin@163.com; 5 College of Civil Engineering, Tongji University, Shanghai 200092, China

**Keywords:** carbon nanotube, elastic conductor, buckle structure, non-enzymatic glucose detection

## Abstract

The increasing demand for wearable glucose sensing has stimulated growing interest in stretchable electrodes. The development of the electrode materials having large stretchability, low detection limit, and good selectivity is the key component for constructing high performance wearable glucose sensors. In this work, we presented fabrication of stretchable conductor based on the copper coated carbon nanotube sheath-core fiber, and its application as non-enzymatic electrode for glucose detection with high stretchability, low detection limit, and selectivity. The sheath-core fiber was fabricated by coating copper coated carbon nanotube on a pre-stretched rubber fiber core followed by release of pre-stretch, which had a hierarchically buckled structure. It showed a small resistance change as low as 27% as strain increasing from 0% to 500% strain, and a low resistance of 0.4 Ω·cm^−1^ at strain of 500%. This electrode showed linear glucose concentration detection in the range between 0.05 mM and 5 mM and good selectivity against sucrose, lactic acid, uric acid, acrylic acid in phosphate buffer saline solution, and showed stable signal in high salt concentration. The limit of detection (LOD) was 0.05 mM, for the range of 0.05–5 mM, the sensitivity is 46 mA·M^−1^. This electrode can withstand large strain of up to 60% with negligible influence on its performance.

## 1. Introduction

Stretchable conductors having stable resistance and high conductivity are key components for wearable electronics [1,2,3,4,5,6]. An efficient way to fabricate stretchable conductors is to use a conducting layer with buckled structure. Such a structure is prepared by coating conductive layer over a pre-stretched elastomeric polymer substrates followed by relax of the prestrain [7,8,9,10,11,12]. During stretching the buckled surface layer fattens without broken and therefore kept resistance constant. The generally used material for the surface conducting layer is metal or semiconductor film, carbon nanotubes, graphene, or conducting polymer fibers. One of the limitations of using metal film is that the buckled metal film based stretchable conductor can only withstand very limited stain range (e.g., 10% for gold film coated on PDMS) [13]. By using buckled silver nanowires or carbon nanotubes as the conducting layer, the resulting stretchable conductors can have an available strain range less than 100% [14,15]. Recently, forest drawn super aligned carbon nanotube sheets (SACNSs) have been used to fabricate the buckled conducting surface layer of the stretchable conductors and the available strain can be improved to 400% [16,17,18]. In our recent work, we have prepared hierarchically buckled structure using SACNS and realized a resistance change less than 5% for over 1000% strain, pushing the stretchability to a record value with obtaining stable resistance [19,20]. As the conductivity of carbon is relatively low, one limitation of such stretchable conductors is that they are suitable for applications that require low conductivity. For example, for constructing wearable glucose sensors that can be used for real time monitoring the glucose concentration, a stretchable electrode with high conductivity and stable resistance is required.

Wearable glucose sensors can measure the glucose concentration in body fluid such as sweat, saliva, and tears [21,22,23]. The normal blood sugar level is in the range of about 4.4 to 6.1 mM [24], and the glucose concentration in sweat was about 1% of that in blood [25]. This means that a detection limit of glucose around 0.05 mM is required for sweat glucose detection. This detection limit of glucose concentration in sweat is much lower than that in blood. Moreover, to provide real time and on-body glucose monitoring, flexibility and stretchability of the electrode is required. Because there is considerable deformation in the skin during people’s movement (e.g., 60% in elbow), high stretchability is preferred [26]. Furthermore, the amperometric response of the electrode was generally interfered by endogenous electro-active chemicals, including sucrose, acrylic acid (AA), lactose, and uric acid (UA). Although these chemicals only presented at much lower concentration than glucose in the body fluids, they may also cause interference for glucose sensing. Therefore, fabrication of the glucose sensing electrode showing high stretchability, a low detection limit, and selectivity is required for high performance non-invasive on body glucose monitoring.

There are two types of electrodes for glucose sensing: enzymatic and non-enzymatic. Enzymatic electrodes have been proven successful in detecting glucose concentrations [27,28,29,30,31]. However, they are limited by the instability of the enzyme, which is easily influenced by pH, temperature, humidity, and oxygen [32]. The non-enzymatic electrodes for glucose detection use metal, metal oxide, carbon nanomaterials, and the combinations of the above two or more species as active materials. During glucose detection, the glucose molecules bind to the electrode and change its electrochemical environment, which serves as an electrochemical catalyst for oxidation of the glucose molecules [5,33]. Compared to the enzymatic counterparts, the non-enzymatic electrodes show much higher stability [34,35,36,37,38,39,40,41]. Recently, there has been rapid development of flexible and stretchable non-enzymatic electrodes, including single metal or metal oxide (Au, Ag [42,43], Pt, Ni [44], NiO [41,45,46], Cu, CuO [47]), bimetallic components (Ag/Cu [48], Pd/Pt [49]), and carbon nanomaterials functionalized with metals [27], as listed in Table 1. Although these recently-developed non-enzymatic electrodes show properties including flexibility or stretchability, low detection limit, and anti-interference, it is still a challenge to prepare a non-enzymatic electrode with the combination of high strain, low detection limit, and selectivity.

In our recent work, by using buckled Pt-coated CNTS based sheath-core conductors, we showed glucose detection under large deformation up to 45% with stable performance. This stretchable electrode showed linear relationship of current with glucose concentration between 0.67 mM and 10.5 mM in phosphate buffer saline (PBS) solution [26]. Here, our strategy is to further modify the above CNTS based stretchable electrode to obtain lower detection limit and introduce selectivity for glucose detection.

There have been several important studies to decrease the detection limit and minimize the interference in glucose sensing caused by the electroactive species in blood fluid [25,53,54,55]. One important strategy is to use Cu and CuO nanoparticles functionalized carbon nanomaterials such as carbon nanotubes, graphene, and carbon black [26,56,57,58,59,60]. This is possibly because of the combination of high catalytic activity of Cu/CuO nanoparticles for glucose oxidation and high electrochemical activity and conductivity of the carbon nanomaterials. Up to now, there has not been a report about fabrication of highly stretchable copper coated carbon nanomaterials used for glucose detection.

Based on these considerations, in this paper we fabricated highly stretchable conductors based on copper coated carbon nanotube sheath-core fibers, and characterized their applications in non-enzymatic glucose sensing electrode with high stretchability, low detection limit, and selectivity. The sheath-core conductor was fabricated by coating copper coated carbon nanotube sheet on a pre-stretched rubber fiber core followed by release of pre-stretch, which showed a hierarchically buckled structure. It showed a small resistance change as low as 27% as strain increasing from 0% to 500% strain, and a low resistance of 0.4 Ω cm^−1^ at 500% strain. In a proof of principle study, this electrode showed linear glucose concentration detection in the range between 0.05 mM and 5 mM and good selectivity against sucrose, lactose, uric acid, and acrylic acid in phosphate buffer saline solution, and showed stable signal in high salt concentration. This electrode can withstand large strain of up to 60% with negligible influence on its performance. Our results push the sheath-core fiber technology for precise measurements of glucose concentration to a much closer step to real applications.

## 2. Materials and Methods

The CNTS used in this work was drawn from spinnable carbon nanotube forest (~6 walls, ~10 nm in diameter, and ~350 μm high) grown by chemical vapor deposition (CVD) method. The rubber contains a mixture of styrene-(ethylene-butylene)-styrene (SEBS, G-1651H, Kraton, Houston, TX, USA) and liquid wax (Marcol 82, ExxonMobil, Irving, TX, USA) with a weight ratio of 1/5.

Fabrication of copper coated CNTS (Cu*_t_*-CNTS) is as follows, where *t* means the thickness of the copper layer. The Cu*_t_*-CNTS was prepared by coating a copper thin layer on the CNTS via magnetron sputter deposition. The samples with different copper thickness were prepared using a Cu target with 8-cm diameter and purity of 99.99%. The holder containing CNTS was rotated during deposition of Cu in order to obtain uniform Cu layer on the CNTS. The distance between the Cu target and the CNTS was 15 cm. A copper plate cooled by water was used to mount the cathode. A base pressure of 1.8 × 10^−3^ Pa in the chamber was realized before deposition of Cu. High purity argon was used as the working gas and the pressure during Cu deposition was 0.6 Pa. The copper thickness was determined using the Alpha-Step 500 surface profiler for the metal film deposited on the glass slide. The deposition rate of copper was calculated to be 1.4 nm·min^−1^, by dividing the film thickness with time.

The fabrication of copper coated CNTS based stretchable conductive fibers (Cu*_t_*-CNTS@fibers) was based on a pre-stretch/release method. Briefly, we stretched the rubber fiber of 2 mm in diameter to a fabrication strain (800% if not specified). Then a thin layer of rubber about 3 μm was coated on the pre-stretched rubber fiber in order to improve the adhesion of Cu*_t_*-CNTS on rubber. Then Cu*_t_*-CNTS supported on a frame was attached on the surface of the pre-stretched rubber fiber coated with a rubber thin layer. An important thing was that the Cu*_t_*-CNTS was aligned with the stretching direction of rubber fiber. After rolling Cu*_t_*-CNTS layers, the Cu*_t_*-CNTS was densified onto the rubber surface by dropping ethanol (98%). Then pre-stretched rubber fiber was slowly relaxed to produce the buckled (Cu*_t_*-CNTS)*_m_*@fibers after ethanol evaporation.

Characterization of Cu*_t_*-CNTS and (Cu*_t_*-CNTS)*_m_*@fibers: The electrical resistance was measured by using a two-probe method via a Keithley SourceMeter modeled 2400 in N_2_ atmosphere. The scanning electron microscope (SEM) micrographs were taken on a Nova NanoSEM450 field emission SEM.

Glucose concentration measurements using the (Cu*_t_*-CNTS)*_m_*@fiber electrode: A three-electrode system was used to measure the amperometric response of glucose concentration using (Cu*_t_*-CNTS)*_m_*@fibers, including a (Cu*_t_*-CNTS)*_m_*@fiber as the working electrode, a Pt wire as the counter electrode, and an Ag/AgCl (KCl-saturated) electrode as the reference electrode, respectively. For all experiments, PBS solution (NaCl: 137 mM, KCl: 2.7 mM, Na_2_HPO_4_: 10 mM, KH_2_PO_4_: 1.8 mM, pH 7.4) and the analyte solutions were freshly prepared. Cyclic voltammograms (CV) of the (Cu_13nm_-CNTS)_3_@fiber electrode and the CNTS_3_@fiber electrode in PBS solution were obtained at different strain, using a scan rate of 100 mV s^−1^ unless otherwise mentioned. The CV curves were obtained at different applied potentials in PBS solution by dropwise addition of glucose and other interferents under magnetically stirred conditions. By adding one drop of glucose, the glucose concentration increased by 0.05 mM in the detection solution, and the applied potentials were +0.25 V, +0.35 V, +0.5 V. Amperometric current–time data were collected under magnetically stirring at a constant applied potential. Repeatability was characterized by measuring the relative amperometric response of the electrode to addition of 1 mM glucose into PBS solution over a period of 72 h with interval of 12 h. The electrode was taken out, washed thoroughly with H_2_O, and put in the ambient condition between the two measurements. After the current curve reached a steady state, the solutions of analyte and interferents were injected. Here, nine samples (three samples from three separate batches) were used. Electrochemical measurements were performed on a CH Instruments (CHI) 660C electrochemical analyzer, and the data were analyzed using the CHI software.

## 3. Results and Discussion

### 3.1. Electrical Properties of (Cu_t_-CNTS)_m_@fibers

**Figure 1:**
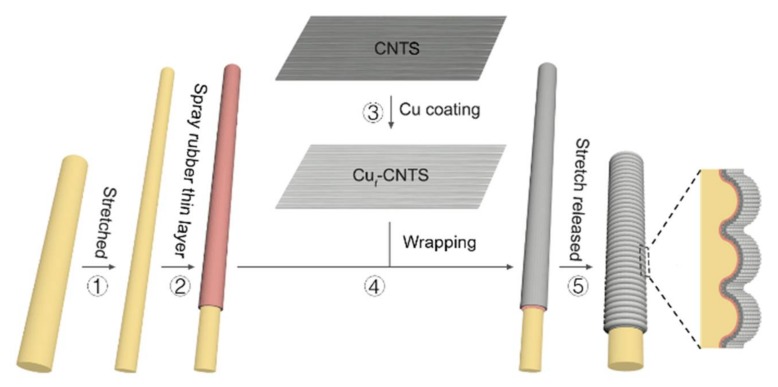
Schematic demonstration of fabrication of (Cu*_t_*-CNTS)*_m_*@fibers. The numbers indicate the fabrication steps.

The CNTS were drawn from a CNT forest which was grown by using a CVD method, and the carbon nanotube bundles were aligned in the drawing direction of the CNTS. The diameter of the individual nanotubes was about 10 nm, and the CNTS was a continuous sheet that was formed by interconnection of the carbon nanotube bundles. The thickness of the as-drawn CNTS sheet was 18 μm [61]. The CNTS showed a highly porous and loose aerogel structure, which allowed the magnetron sputtered species to easily pass through the nanotube sheets and disperse over the CNT bundles, as shown in Figure 1.

**Figure 2:**
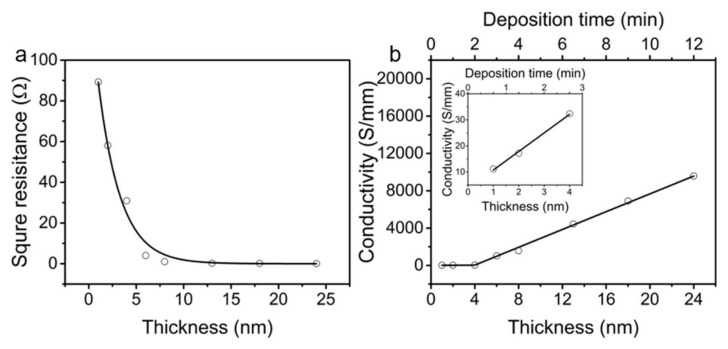
(**a**) The square resistance of the Cu*_t_*-CNTS as a function of Cu thickness. (**b**) The conductivity of the Cu*_t_*-CNTS as a function of deposition time and thickness of Cu. The inset showed conductance of Cu*_t_*-CNTS as a function of deposition time and thickness of Cu for deposition time shorter than three minutes.

Magnetron sputter deposition was widely used to deposit thin metal films for diverse applications because of its high rate, ease of scaling, and high quality of the deposited film [62]. Here, the copper was deposited on the CNTS using magnetron sputter deposition to improve the conductivity and electro-catalytic activity. The sheet resistance for an as-produced single CNTS was ~1000 Ω per square (equal to 1 S mm^−1^) in the drawing direction, which was relatively high for applications in interconnects for electric circuits and sensing electrodes. After copper deposition, we tested the resistance in N_2_ atmosphere. The sheet conductance increased from 11.2 S mm^−1^ to 32.3 S mm^−1^ with the deposition time of copper increasing from 40 s to 160 s, corresponding to an average conductance increasing rate of 0.18 S mm^−1^ s^−1^. With further increasing the deposition time from 160 s to 700 s, the sheet conductance increased dramatically from 32.3 S mm^−1^ to 9577 S mm^−1^, corresponding to an average conductance increasing rate of 17.7 S mm^−1^ s^−1^ (Figure 2).

**Figure 3:**
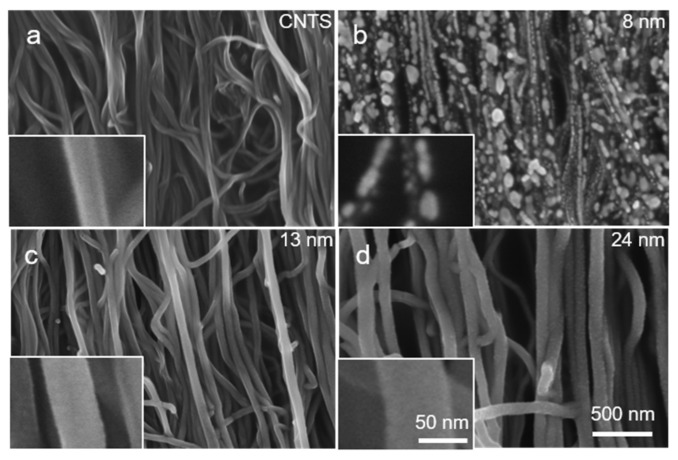
(**a**–**d**) The SEM images of the Cu*_t_*-CNTS with different thickness of copper.

Figure 3 showed the SEM images of the surface for Cu*_t_*-CNTS samples with different deposition time. For deposition time from 60 s to 240 s, discrete Cu nanoparticles were observed on the carbon nanotube surface. For these samples, although the conductance increased with increasing Cu deposition time, the Cu nanoparticles with an average thickness of 8 nm did not form a continuous pathway (Figure 3b). Therefore, the conductance increased relatively slow with increasing the deposition time. With further increasing the deposition time to 400 s (thickness of Cu layer is about 13 nm), uniform surface was observed (Figure 3c). The copper nanoparticles contacted with one another and formed continues pathway, and therefore the conductance should be dominated by the copper sheath coated over the carbon nanotube bundles, which should be responsible for the rapid increase in the conductance with increase of the thickness of Cu layer. The thickness of Cu coating was obtained by measuring the thickness of the deposited film on a glass slide. For the convenience of nomenclature, the average thickness of the Cu*_t_*-CNTS samples with discrete Cu nanoparticles was also normalized to those having continuous Cu layer by counting the deposition time, as shown in Figure 3. It is worth noticing that the actual thickness of the Cu layer might be smaller than that of the film coated on the glass slide because the bundles of the CNT were not continuous so that some Cu atoms may pass through the gaps between the CNT bundles. From the SEM image of the surface of Cu_24nm_-CNTS (Figure 3d), we can clearly see that a continuous Cu layer was coated on the CNT bundle. The remarkable conductance and highly alignment architecture of the Cu*_t_*-CNTS allowed the fabrication of superelastic sheath–core conductors [55].

We fabricated the sheath-core (Cu*_t_*-CNTS)*_m_*@fibers by using a similar method to our previous work for CNTS based sheath-core fiber [26,55,63,64], except that a very thin layer of rubber was coated over the pre-stretched rubber fiber in order to improve the adhesion of the Cu*_t_*-CNTS layer on the rubber fiber core. Briefly, we stretched a rubber fiber to the fabrication strain, and then spayed with a thin layer of rubber (3 μm) over the rubber fiber core. We then coated layers of Cu*_t_*-CNTS on the pre-stretched rubber fiber. It should be noted that the orientation direction of the Cu*_t_*-CNTS was the same as the stretch direction of the rubber fiber. Ethanol was used to densify the porous Cu*_t_*-CNTS on the rubber fiber, and then we released the applied strain of the rubber fiber, as shown in Figure 1. The fiber diameter was typically 2 mm, and the fabrication strain was typically 800% if not specified.

**Figure 4:**
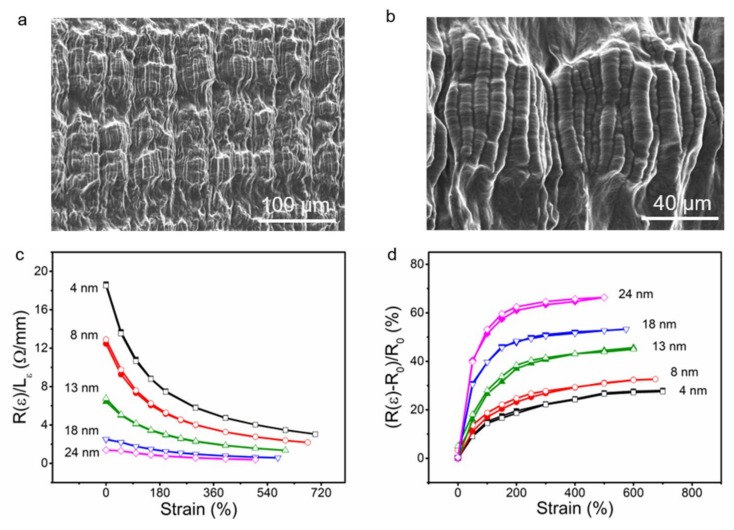
Low- (**a**) and high- (**b**) magnification SEM images of the (Cu_13nm_-CNTS)_3_@fiber at 0% strain. The circumferential direction is vertical and the length direction is horizontal. The resistance normalized to length (**c**) and percent resistance change (**d**) for (Cu*_t_*-CNTS)_3_@fibers as a function of applied strain with different thickness of copper layer. The solid symbols represented strain increase and the open symbols represented subsequent strain release. The fabrication strain was 800% and the fiber diameter was 2 mm for (**a**–**d**).

Before characterization, the Cu*_t_*-CNTS sheath core fibers were trained for five cycles by repeatedly stretching the fiber to the fabrication strain and fully relaxing. We choose the fabrication strain of the (Cu*_t_*-CNTS)*_m_*@fibers as the maximum length because irreversible plastic deformation of the Cu*_t_*-CNTS would occur if when the (Cu*_t_*-CNTS)*_m_*@fibers are beyond the fabrication strain. Figure 4a,b showed the SEM images of the (Cu_13nm_-CNTS)_3_@fiber, it can be seen that hierarchical buckling in the fiber length direction and necking in the circumferential direction occurred for the Cu_13nm_-CNTS conducting layer. The formation of buckling structure in the length direction and necking in the circumferential direction is because the rubber material is non-compressive (the Poisson’s ratio of rubber is about 0.5). During the relaxation of the pre-strained rubber fiber, the fiber length decreased by *L* times and the fiber diameter increased by *L*^−1/2^ times accordingly. This length decrease caused buckle formation and the diameter increase caused stretch of the copper coated carbon nanotube sheath and then induced necking [65]. This two orders of hierarchically buckled structure of (Cu_13nm_-CNTS)_3_@fiber was different from the buckled structures of the CNTS*_m_*@fibers, where one order of buckled structure was observed if the number of CNTS layer was smaller than five [55]. Because a rubber thin layer was applied for adhesion before deposition of the Cu*_t_*-CNTS conducting layer during fabrication of CNTS*_m_*@fibers, the two orders of hierarchical buckling structure of the (Cu_13nm_-CNTS)_3_@fiber may be a consequence of the buckling of the sprayed rubber adhesion layer superimposed with the buckling of CNTS layer during relaxation of the pre-strain of rubber fiber core [26]. The final (Cu_13nm_-CNTS)_3_@fiber showed smaller available strain range than the fabrication strain because of the mechanical confinement of the buckled Cu*_t_*-CNTS sheath layer. The available elastic strain range of the (Cu_13nm_-CNTS)_3_@fiber prepared in this paper could be up to 600%. CNT bundles coated with copper layer generated a metal-carbon hybrid conducting material with structural anisotropy. Because of the flexibility of the CNTS skeleton, the hybrid material should possess more flexibility compared to the metal thin film. Furthermore, the copper coated CNT bundles are continuous in stretch/release direction of the rubber fiber and discontinuous in the circumferential direction of the (Cu*_t_*-CNTS)*_m_*@fibers. Therefore, it can accommodate circumferential expansion during large strain relaxation (up to 600% in our case) of the rubber fiber core. This is advantageous compared to the thin metal film based stretchable conductors, which can only withstand a limited strain of up to 22% for obtaining stable resistance and cracks would occur perpendicular to the stretch–release direction [66,67].

Increasing the copper thickness in the Cu*_t_*-CNTS hybrid layer resulted in decrease of resistance of the (Cu*_t_*-CNTS)*_m_*@fibers. Figure 4c showed the results of length normalized resistance (*R*(ε)/*L_ε_*) as a function of tensile strain (ε) of the (Cu*_t_*-CNTS)_3_@fibers having different Cu thickness (*t*), where *R*(ε) and *L*(ε) were the resistance and length at strain (ε) of the (Cu*_t_*-CNTS)*_m_*@fibers. For the (Cu*_t_*-CNTS)_3_@fibers at 0% strain, *R*(0)/*L*_0_ decreased from 19 Ω·cm^−1^ to 1.4 Ω·cm^−1^ as *t* increased from 4 nm to 24 nm. For the (Cu*_t_*-CNTS)_3_@fibers in the stretched state, *R*(500%)/*L*_500%_ decreased from 4 Ω·cm^−1^ to 0.4 Ω·cm^−1^ as *t* increased from 4 nm to 24 nm, as shown in Figure 4c. *R*(ε)/*L_ε_* of the (Cu*_t_*-CNTS)_3_@fibers decreased monotonically with increase of strain (ε). For example, *R*(ε)/*L_ε_* of (Cu_4nm_-CNTS)_3_@fiber decreased from 19 Ω·cm^−1^ to 3 Ω·cm^−1^ as strain increased from 0% to 700%; *R*(ε)/*L_ε_* of (Cu_24nm_-CNTS)_3_@fiber decreased from 1.4 Ω·cm^−1^ to 0.4 Ω·cm^−1^ as strain increased from 0% to 500%.

Figure 4d showed the percent resistance change ((*R*(ε)-*R*_0_)/*R*_0_) of the (Cu*_t_*-CNTS)_3_@fibers as a function of copper thickness (*t*), where *R*_0_ is the resistance at 0% strain. It can be seen that the (*R*(ε)-*R*_0_)/*R*_0_ values of (Cu*_t_*-CNTS)_3_@fibers were 27, 31, 44, 53, and 66% for *t* = 4, 8, 13, 18, and 24 nm, respectively, as strain increased from 0% to 500%. This indicated that the resistance of the (Cu*_t_*-CNTS)*_m_*@fibers decreased with increasing the thickness of the copper layer on the CNTS, and the resistance got less stable with strain increase. This is possibly because the flexibility of the Cu*_t_*-CNTS layer decreased with increase of the copper layer.

### 3.2. Stretchable Non-Enzymatic Electrode for Glucose Sensing Based on the (Cu_t_-CNTS)_m_@fibers

**Figure 5:**
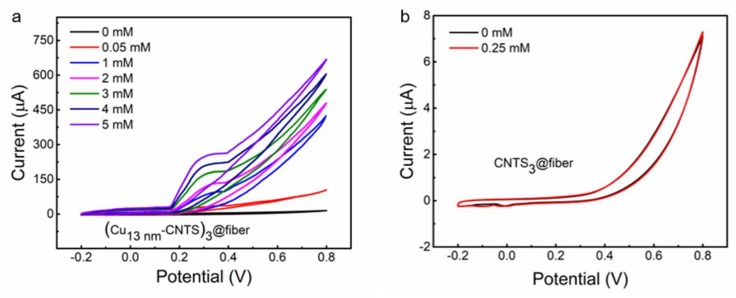
(**a**,**b**) Cyclic voltammograms of the (Cu_13nm_-CNTS)_3_@fiber electrode at different glucose concentrations (0, 0.05, 1, 2, 3, 4, and 5 mM) (**a**) and the CNTS_3_@fiber electrode (**b**) in PBS solution at 0% strain and 100 mV s^−1^.

We then characterized the (Cu*_t_*-CNTS)*_m_*@fibers for used in non-enzymatic glucose detection. We constructed a three-electrode system including a working electrode using (Cu_13nm_-CNTS)_3_@fiber, a reference electrode using Ag/AgCl electrode, and a counter electrode using a platinum wire. Figure 5a,b showed CV of (Cu_13nm_-CNTS)_3_@fiber electrode in PBS solution at 0% strain with a scan rate of 100 mV s^−1^, using CNTS_3_@fiber electrode as the control.

There were no substantial peaks observed for the CV of both (Cu_13nm_-CNTS)_3_@fiber and CNTS_3_@fiber without glucose addition, except for the tail-shaped peak occurred at +0.65 V, which corresponded to the electrolysis of H_2_O molecules, as shown in Figure 5a,b, respectively. Figure 5a showed that by adding different concentrations of glucose, the (Cu_13nm_-CNTS)_3_@fiber showed peaks of current appearing at +0.35 V and +0.65 V versus the Ag/AgCl electrode. The shoulder peak occurred at +0.35 V was ascribed to the glucose oxidation on copper. This indicated high catalytic capacity of copper coated CNTS for glucose oxidation to gluconic acid. In comparison, no new peaks were observed by the addition of glucose on the CNTS_3_@fiber electrode system as shown in Figure 5b, indicating that the pure CNTS can hardly be used for glucose sensing at the same electrochemical conditions.

It was reported that the amperometric signal of an electrochemical electrode was highly dependent on the applied potential. Below we studied the influence of applied potential on the amperometric signals of our (Cu_13nm_-CNTS)_3_@fiber electrode. These experiments were carried out on (Cu_13nm_-CNTS)_3_@fiber electrode at 0% strain, by dropwise addition of glucose into PBS solution at interval of 50 s by applying potential from +0.25 V to +0.50 V. The dropwise addition of glucose resulted in step-wise increase of glucose concentration.

**Figure 6:**
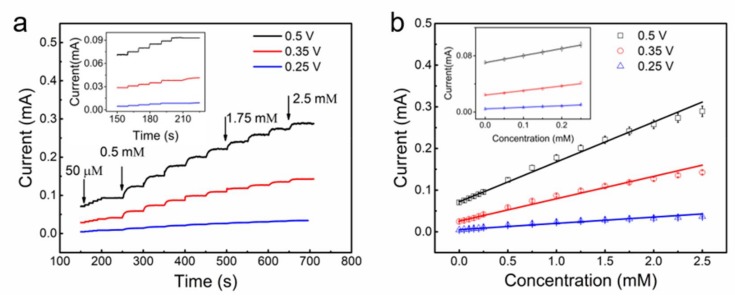
(**a**) Current as a function of time of the (Cu_13nm_-CNTS)_3_@fiber electrode at different applied potentials in PBS solution by dropwise addition of glucose. (**b**) The calibration curve of the current as a function of concentration of the (Cu_13nm_-CNTS)_3_@fiber electrode at different applied potential, nine samples (three samples from three separate batches) are used here to get the average values. For (**a**,**b**), by adding one drop of glucose, the glucose concentration increased by 0.05 mM for the low concentration range and 0.5 mM for the high concentration range, and the applied potentials were +0.25, +0.35, and +0.5 V. Insets: current as a function of time for low concentration range of glucose for both (**a**,**b**).

Figure 6a showed the current was very low at an applied potential of +0.25 V, and the current of (Cu_13nm_-CNTS)_3_@fiber electrode increased remarkably at the applied potentials of +0.35 V and +0.5 V. Figure 6b showed the dependence of current on the (Cu_13nm_-CNTS)_3_@fiber electrode on glucose concentration from 0.05 mM to 2.5 mM at different applied potentials, with 0.05 mM and 0.5 mM for low and high glucose concentration regions. The calibration curve of the current showed linear response with increase of the glucose concentration at the investigated potentials from nine samples (three samples from three separate batches). It is noticeable that the application of potential of +0.35 V vs. Ag/AgCl on the working electrode was close to the voltage corresponding to glucose oxidation, where the current peak was observed from the CV curve of the (Cu_13nm_-CNTS)_3_@fiber. At the working potential of +0.35 V, the sensitivity of the (Cu_13nm_-CNTS)_3_@fiber electrode at 0% strain was calculated to be ~46 mA·M^−1^ for the investigated glucose concentration range of 0.05–5 mM, by dividing the detected current change with glucose concentration change. The limit of detection (LOD) is 0.05 mM. By considering the glucose oxidation peak around +0.35 V, as well as the reasonable electrochemical current and linear relationship were obtained at this potential, +0.35 V was applied as the working potential in the following investigations for stretchability, selectivity, and stability tests.

**Figure 7:**
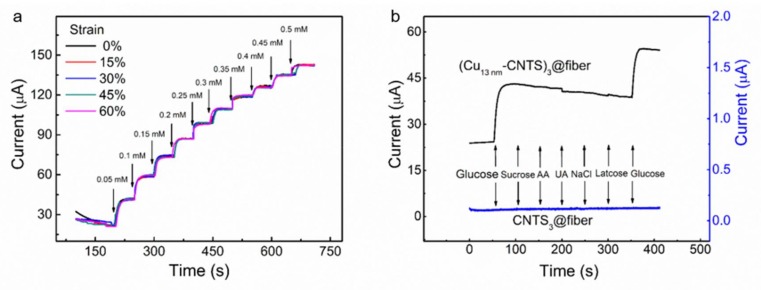
(**a**) Current as a function of time of the (Cu_13nm_-CNTS)_3_@fiber electrode at different strain with dropwise addition of glucose in PBS solution at 50 s interval. By adding one drop of glucose, the glucose concentration increased by 0.05 mM in the detection solution, and the detection potential was +0.35 V. (**b**) Current as a function of time of the (Cu_13nm_-CNTS)_3_@fiber electrode and CNTS_3_@fiber electrode with addition of glucose and interferents as indicated into PBS solution. By adding one drop of glucose, the glucose concentration increased by 0.05 mM in the detection solution, and by adding one drop of interferents, the corresponding concentration also increased as follows: sucrose (0.008 mM), AA (0.008 mM), UA (0.008 mM), NaCl solution (150 mM), and lactose (0.008 mM). The applied potential was +0.35 V.

Figure 7a showed current during stepwise drop addition of glucose for the (Cu_13nm_-CNTS)_3_@fiber electrode at strain of 0, 15, 30, 45, and 60%, respectively. It can be seen that the detected current almost unchanged for the investigated glucose concentration range from 0.05 mM to 0.5 mM for the investigated strain level. This indicated that our (Cu*_t_*-CNTS)*_m_*@fiber electrode showed good stability for non-enzymatic glucose detection under large deformations.

To quantitatively investigate the stability of glucose detection during deformation, we calculated the quality factor for glucose sensing (*Q*_gs_) of the (Cu_13nm_-CNTS)_3_@fiber electrode for the strain range from 0% to 60%. The *Q*_gs_ was calculated by the Equation (1)

*Q*_gs_ = ((*I*(ε) − *I*_0_)/*I*_0_)/(ε − 0%) (1)

where *I*(ε) and *I*_0_ are the current at strain of ε and 0%, respectively. The calculated value of *Q*_gs_ of the (Cu_13nm_-CNTS)_3_@fiber electrode was about 30 for the strain range from 0% to 60%, which was higher than our previously investigated glucose sensing system of platinum coated CNTS based stretchable electrode (*Q*_gs_ = 22 for strain range from 0% to 45%). This high stability during large deformation for glucose sensing of the (Cu*_t_*-CNTS)*_m_*@fiber electrode made it a promising candidate for constructing wearable sensors for non-enzymatic glucose monitoring during extreme on-body deformation.

**Figure 8:**
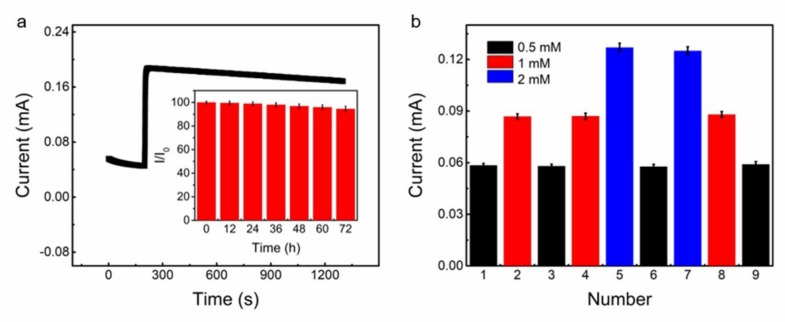
(**a**) Amperometric response of the (Cu_13nm_-CNTS)_3_@fiber electrode by addition of 1 mM glucose into the PBS solution and stored for a long period of running time (1000 s), The inset is the repeated measurements of the calibration curve of the relevant amperometric signal of the sensor electrode with an interval of 12 h in PBS solution during 72 h, with 1.0 mM glucose at +0.35 V. (**b**) The calibrated consecutive measurements of the (Cu_13nm_-CNTS)_3_@fiber electrode in glucose solution with different concentrations. For (**a**,**b**), nine samples (three samples from three separate batches) are used here to get the average values.

We then characterized the selectivity of our stretchable (Cu*_t_*-CNTS)*_m_*@fiber electrode against interferents—including sucrose, AA, lactose, and UA—as well as in high salt concentration. These chemicals were generally present in human physiological fluids and blood serum accompanying with glucose. Although the glucose concentration in human blood in normal physiological conditions was much higher (about 30 times) than that of the sucrose, AA, lactose, and UA [57], we used a higher level of interferent species to test the selectivity of the (Cu*_t_*-CNTS)*_m_*@fiber electrode. We successively added glucose (0.05 mM, the final concentration, and the same for the following chemicals) and sucrose (0.008 mM), AA (0.008 mM), lactose (0.008 mM), NaCl (150 mM), and UA (0.008 mM) into PBS solution by dropwise addition of glucose and the relevant interfering species at an applied potential of +0.35 V, and the results were shown in Figure 7b. With addition of glucose, we can clearly see the amperometric response, while by adding sucrose, AA, lactose, and UA, the (Cu_13nm_-CNTS)_3_@fiber electrode did not show any significant current increase. These results indicated that (Cu_13nm_-CNTS)_3_@fiber electrode could be used as a highly selective electrode for glucose detection. Furthermore, the (Cu_13nm_-CNTS)_3_@fiber electrode also showed negligible change of the response by addition of high concentration of NaCl. We then studied the stability and long-term repeatability of the (Cu_13nm_-CNTS)_3_@fiber electrode from nine samples (three samples from three separate batches). Figure 8a showed that there was negligible signal loss in current over 1000 s of running time, indicating good stability of the electrode. The inset in Figure 8a showed the repeated measurements of the relative amperometric response of the electrode to addition of 1 mM glucose into PBS solution over a period of 72 h with interval of 12 h. The final relative amperometric response was 94.5% of the initial value after 72 h, indicating good repeatability and long-term stability of the electrode. Compared to the stretchable glucose sensing electrodes [28,29,56], our non-enzymatic electrode based on (Cu*_t_*-CNTS)*_m_*@fibers showed simultaneous combination of high stretchability (60%), high sensitivity (46 mA·M^−1^), low limit of detection (0.05 mM), high selectivity, and the nature of non-enzymatic detection. Figure 8b showed the consecutive measurements of the (Cu_13nm_-CNTS)_3_@fiber electrode in glucose solution from low concentration to high concentration, and then back to low concentrations, indicating the good reversibility of the electrode.

## 4. Conclusions

In summary, we prepared highly stretchable (Cu*_t_*-CNTS)*_m_*@fiber conductor by using buckled copper coated CNTS sheath and rubber fiber core, and demonstrated it as a non-enzymatic glucose sensing electrode. This (Cu*_t_*-CNTS)*_m_*@fiber showed a low resistance of 0.4 Ω·cm^−1^ at strain of 500%, and little resistance change of 27% with strain increasing from 0% to 500%. The amperometric response of the (Cu*_t_*-CNTS)*_m_*@fibers electrode for glucose detection was stable for a large strain range up to 60%, with a low detection limit of 0.05 mM, high sensitivity of 46 mA·M^−1^, and selectivity against sucrose, AA, lactose, UA, and high salt concentrations. The (Cu*_t_*-CNTS)*_m_*@fiber could be used as a promising candidate electrode for wearable on-body glucose monitoring.

## Figures and Tables

**Table 1:** Comparison of the non-enzymatic stretchable and flexible glucose sensing electrodes with selectivity against interferents.

Electrode	V_App._^1^ (V)	LOD ^2^ (µM)	Strain (%)	Selectivity Test	Reference
Graphene/CNT ^3^/Ionic Liquid	+0.2	100	Flexible	DA ^4^/AA ^5^/UA ^6^/NaCl	[36]
3D CuO Nanowire Arrays	+0.35	20	Flexible	AA/Lactose/Maltose	[45]
PtAu-MnO_2_/Graphene Paper	0	20	Flexible	DA/AA/UA	[46]
Ni/VCNTs ^7^/Graphene	+0.5	30	Flexible	AA/GA ^8^/XY ^9^/UA	[47]
NiO QDs ^10^-ZnO NRs ^11^/Polyimide	n/a	26	Flexible	DA/AA/UA/cholesterol	[48]
Au Nanoparticles/Polyaniline/Carbon Cloth	0	12.6	Flexible	AA/UA/AMP ^12^/d-galactose/fructose	[49]
Cu Nanoparticles/RGO ^13^	+0.6	1000	Flexible	AA/UA/DA	[50]
Pt/CNTS ^14^/Rubber	+0.3	660	45%	n/a	[51]
Pt-Graphite	+0.3	4800	75%	AA/UA/DP ^15^/EPI ^16^	[52]
Cu/CNTS/Rubber	+0.35	50	60%	AA/Sucrose/Lactose/UA/NaCl	**This work**

^1^ V_app._: Applied Potential; ^2^ LOD: Limit of Detection; ^3^ CNTS: Carbon Nanotube; ^4^ DA: Dopamine; ^5^ AA: Ascorbic Acid; ^6^ UA: Uric Acid; ^7^ VCNTs: Vertically Aligned Carbon Nanotubes; ^8^ GA: Galactose; ^9^ XY: Xylose; ^10^ NiO QDs: Nickel Oxide Quantum Dots; ^11^ ZnO NRs: Zinc Oxide Nanorods; ^12^ AMP: Adenosine Monophosphate; ^13^ RGO: Reduced Graphene Oxide; ^14^ CNTS: Carbon Nanotube Sheets; ^15^ DP: Docking Protein; ^16^ EPI: Epirubicin.
